# Myoclonus dystonia and muscular dystrophy: ɛ‐sarcoglycan is part of the dystrophin‐associated protein complex in brain

**DOI:** 10.1002/mds.26738

**Published:** 2016-08-18

**Authors:** Adrian J. Waite, Francesca A. Carlisle, Yiumo Michael Chan, Derek J. Blake

**Affiliations:** ^1^ Division of Psychological Medicine and Clinical NeurosciencesMRC Centre for Neuropsychiatric Genetics and Genomics, Cardiff UniversityCardiffUnited Kingdom; ^2^ McColl‐Lockwood Laboratory for Muscular Dystrophy ResearchCarolinas Medical CenterCharlotteNorth CarolinaUSA

**Keywords:** dystonia, DYT11, sarcoglycan

## Abstract

**Background:**

Myoclonus‐dystonia is a neurogenic movement disorder caused by mutations in the gene encoding ɛ‐sarcoglycan. By contrast, mutations in the α‐, β‐, γ‐, and δ‐sarcoglycan genes cause limb girdle muscular dystrophies. The sarcoglycans are part of the dystrophin‐associated protein complex in muscle that is disrupted in several types of muscular dystrophy. Intriguingly, patients with myoclonus‐dystonia have no muscle pathology; conversely, limb‐girdle muscular dystrophy patients have not been reported to have dystonia‐associated features. To gain further insight into the molecular mechanisms underlying these differences, we searched for evidence of a sarcoglycan complex in the brain.

**Methods:**

Immunoaffinity chromatography and mass spectrometry were used to purify ubiquitous and brain‐specific ɛ‐sarcoglycan directly from tissue. Cell models were used to determine the effect of mutations on the trafficking and assembly of the brain sarcoglycan complex.

**Results:**

Ubiquitous and brain‐specific ɛ‐sarcoglycan isoforms copurify with β‐, δ‐, and ζ‐sarcoglycan, β‐dystroglycan, and dystrophin Dp71 from brain. Incorporation of a muscular dystrophy‐associated β‐sarcoglycan mutant into the brain sarcoglycan complex impairs the formation of the βδ‐sarcoglycan core but fails to abrogate the association and membrane trafficking of ɛ‐ and ζ‐sarcoglycan.

**Conclusions:**

ɛ‐Sarcoglycan is part of the dystrophin‐associated protein complex in brain. Partial preservation of ɛ‐ and ζ‐sarcoglycan in brain may explain the absence of myoclonus dystonia‐like features in muscular dystrophy patients. © 2016 The Authors. Movement Disorders published by Wiley Periodicals, Inc. on behalf of International Parkinson and Movement Disorder Society.

Myoclonus dystonia (M‐D) is a combined dystonia syndrome characterized by myoclonic jerks in combination with focal or segmental dystonia.^1^, ^2^, ^3^ Clinical features of the disorder often develop during childhood or early adolescence, initially with myoclonus accompanied by mild dystonia. Alcohol ingestion is frequently noted to temporarily relieve myoclonus; however, sustained use frequently leads to alcohol dependence.^4^ Psychiatric abnormalities such as obsessive‐compulsive disorder, depression, anxiety, and panic attacks have also been reported in many M‐D families.^5^, ^6^ Mutations in the gene encoding ɛ‐sarcoglycan (*SGCE*, DYT11) have been found in approximately 30%‐50% of M‐D cases.^7^ In these families, M‐D is inherited in an autosomal‐dominant pattern with reduced penetrance on maternal transmission because of imprinting of the maternal allele.^7^, ^8^ In addition to *SGCE*, mutations in *KCTD17* (DYT26) have recently been identified in 2 families with autosomal‐dominant M‐D.^9^ A third M‐D locus on chromosome 18 (DYT15) has also been described, although the causative gene remains unidentified.^10^

DYT11 M‐D is caused by loss‐of‐function mutations in *SGCE* that lead to the absence or reduction of the ɛ‐sarcoglycan protein at the plasma membrane.^7^, ^11^, ^12^, ^13^ ɛ‐Sarcoglycan is a member of the sarcoglycan family of transmembrane glycoproteins.^14^, ^15^ Mutations in the genes encoding α‐, β‐, γ‐, and δ‐sarcoglycan cause different limb girdle muscular dystrophies (LGMD2C‐F). The sixth sarcoglycan, ζ‐sarcoglycan, has not been associated with a disease in humans.^16^ Accordingly, *SGCZ* mutations have also been excluded in a cohort of *SGCE* mutation‐negative dystonia patients.^17^ The sarcoglycans form a subcomplex of the larger dystrophin‐associated glycoprotein complex (DGC) in skeletal muscle and other tissues.^15^, ^18^, ^19^ Immunohistochemical analyses of muscle biopsies from LGMD2C‐F patients show that in most cases, deficiency of the mutant sarcoglycan results in concomitant reduction or absence of the other sarcoglycans at the sarcolemma.^20^, ^21^ Although the mechanism controlling the membrane trafficking of the sarcoglycan complex is not fully understood, endoplasmic reticulum–associated degradation has been shown to participate in the quality‐control pathway for mutant sarcoglycans.^12^, ^22^, ^23^, ^24^, ^25^

The sarcoglycans form a heterotetrameric assembly at the cell membrane consisting of a βδ‐sarcoglycan core with additional incorporation of α/ɛ‐ and γ/ζ‐sarcoglycan to complete the tetramer.^26^, ^27^, ^28^, ^29^ The αβδγ‐tetramer is thought to predominate in skeletal and cardiac muscle, although the ɛβδγ configuration has also been described.^30^, ^31^, ^32^ Indeed, mice with mutations in the genes encoding both α‐ and ɛ‐sarcoglycan have a more severe muscle phenotype than α‐sarcoglycan‐deficient mice and develop severe cardiomyopathy because of disruption of the cardiac DGC.^33^ Complexes consisting of ɛβδζ‐sarcoglycan have also been described in smooth muscle, Schwann cells, and adipose tissue.^16^, ^26^, ^34^ In contrast to the other sarcoglycans, *SGCE* is also extensively alternatively spliced, producing several tissue‐specific isoforms.^35^, ^36^, ^37^ Brain‐specific isoforms of ɛ‐sarcoglycan result from alternative splicing of exons 11b and 11c, resulting in variable C‐terminal tail sequences corresponding to the isoforms ɛ‐sarcoglycan type 2 and ɛ‐sarcoglycan type 3.^35^, ^36^, ^38^ In this study we will refer to the ubiquitous ɛ‐sarcoglycan isoform as ɛ‐sarcoglycan‐1 and the brain‐specific ɛ‐sarcoglycan isoform containing exon 11b as ɛ‐sarcoglycan‐2.

Although the role of *SGCE* mutations in the genetic etiology of M‐D is well established, surprisingly little is known about the function of ɛ‐sarcoglycan in the brain. Paradoxically, M‐D patients with *SGCE* mutations have no apparent muscle pathology despite the participation of ɛ‐sarcoglycan in striated and smooth muscle sarcoglycan complexes.^39^, ^40^ Similarly, there is no published evidence to suggest that LGMD patients have features of dystonia or myoclonus, contrasting with the predominantly neurological presentation of M‐D.^40^ Therefore, to gain further insight in to the role of ɛ‐sarcoglycan in M‐D, we searched for evidence of an endogenous sarcoglycan complex in the brain.

## Materials and Methods

### Molecular Biology

Myc‐tagged ɛ‐sarcoglycan isoforms, FLAG‐tagged human β‐sarcoglycan (wild‐type and T182A, c.544A>G mutant^41^, HA‐tagged δ‐sarcoglycan and myc‐tagged ζ‐sarcoglycan were produced by polymerase chain reaction (PCR) and cloned into either pCI‐neo (Promega) or pCMV‐myc (Clontech). All constructs were completely sequenced before use. The two anti‐ɛ‐sarcoglycan‐2‐specific antibodies (1355 and 1358) were raised in rabbits (CovalAb S.A.S) immunized with a short peptide (NH_2_‐C‐QRFEVNGIPEERKLTEAMSL‐COOH) corresponding to the unique C‐terminus of the brain specific ɛ‐sarcoglycan conjugated to keyhole limpet hemocyanin. The resultant antisera were purified using affinity chromatography against the immunogen coupled to Sulfolink resin (Pierce, Rockford, IL). In addition to the peptide antisera, esg‐4990 was raised against a fusion protein corresponding to the entire cytoplasmic domain of ɛ‐sarcoglycan and includes the brain‐specific C‐terminus (ɛ‐sarcoglycan‐2). Other ɛ‐sarcoglycan antibodies have been described elsewhere.^12^, ^13^, ^26^ Monoclonal antibodies NCL‐b‐DG, NCL‐b‐SARC, and NCL‐dys2 were purchased from Leica Biosystems. The ɛ‐ and ζ‐sarcoglycan‐specific peptide polyclonals (ANA‐ɛ and ANA‐ζ) were described by Cai and colleagues, whereas the δ‐sarcoglycan‐specific polyclonal was kindly supplied by Dr. Vincenzo Nigro.^26^, ^42^ Anti‐FLAG M2 and the anti‐HA antibodies were purchased from Sigma Aldrich and Covance, respectively, whereas the anti‐myc 9E10, MANDAG2, and MANDRA1 antibodies were obtained from the Developmental Studies Hybridoma Bank, University of Iowa.^43^, ^44^ Western blotting and membrane preparations were performed as described previously.^13^, ^26^

### Cell Culture and Surface Biotinylation

HEK293T cells were cultured and transfected as described previously.^12^, ^13^ Cell surface biotinylation was performed as described by Waite et al.^13^ Briefly, transfected HEK293T cells were incubated with the membrane‐impermeable sulfo‐NHS‐LC‐biotin reagent (Pierce) that reacts with free amine exposed on the cell surface. After 30 minutes of labeling, the reaction was quenched with 50 mM Tris HCl (pH 8.0), and the cells were lysed in NP‐40 buffer (50 mM Tris HCl [pH 7.4]], 150 mM NaCl, 1% [v/v] NP‐40, Roche EDTA‐free protease inhibitors) before immunoprecipitation with esg‐4990‐protein A agarose beads or affinity purification with NeutrAvidin agarose beads (Pierce). Biotinylated proteins were detected using streptavidin conjugates.

### Immunoaffinity Purification (IAP) and Mass Spectrometry

Tissue extracts were prepared in RIPA or digitonin‐containing (1% [v/v]; Merck Chemicals) buffer as described previously.^12^ The lysates were precleared with 200 µL of packed protein A/G agarose beads for 3 hours at 4 °C. The precleared lysate was divided into 2 fractions: one incubated with 30 µL of packed antibody‐conjugated protein A agarose beads, and the other 30 µL of packed RIPA buffer equilibrated protein A/G agarose beads. Samples were incubated under constant rotation for 3‐5 hours or overnight at 4 °C. After extensive washing in RIPA buffer, bound proteins were eluted in 60 µL of 1 × lithium dodecyl sulfate buffer (Invitrogen) or 2 × Laemmli sample buffer (without reducing agent) at 95 °C for 5 minutes. Samples were cooled on ice prior to the addition of DTT to a final concentration of 50 mM. Forty‐microliter aliquots of each eluent were resolved on a 4%‐12% gradient NuPAGE Novex Bis‐Tris gel (Invitrogen), run in 1 × MOPS running buffer (Invitrogen) using an XCell Surelock Mini‐Cell electrophoresis system (Invitrogen). Gels were run for 90 minutes at 150 V before fixing for 15 minutes in 50% (v/v) methanol and 7% (v/v) acetic acid diluted in CHROMOSOLV Plus high‐pressure liquid chromatography (HPLC)‐grade water (Sigma). Gels were washed twice in HPLC‐grade water prior to staining with 20 mL of colloidal Coomassie blue stain (Gelcode Blue Stain Reagent, Pierce) for 2 hours, followed by destaining with HPLC‐grade water. Coomassie‐stained gels were scanned using an Odyssey Infrared Imaging System, and bands of interest were excised and processed for mass spectrometry (Advanced Mass Spectrometry Facility, University of Birmingham). Trypsin digests were carried out on the gel plugs using a Qiagen 3000 robot. The samples were then loaded onto a chip‐based nanoelectrospray system (Advion Biosciences Nanomate), where liquid chromatography/mass spectrometry was performed, nanoelectrospray ionization initiated and ions sprayed into a Thermo Finnigan LTQ‐FT hybrid mass spectrometer. Analysis of the data was carried out using the BioWorks 3.2 program (Thermo Scientific) that utilizes the SEQUEST search algorithm. In addition to the FT‐ICR mass spectrometry, some experiments were performed using a Velos Orbitrap (Thermo Fisher) mass spectrometer.

## Results

### Identification of Sarcoglycans in the Brain

**Figure 1:**
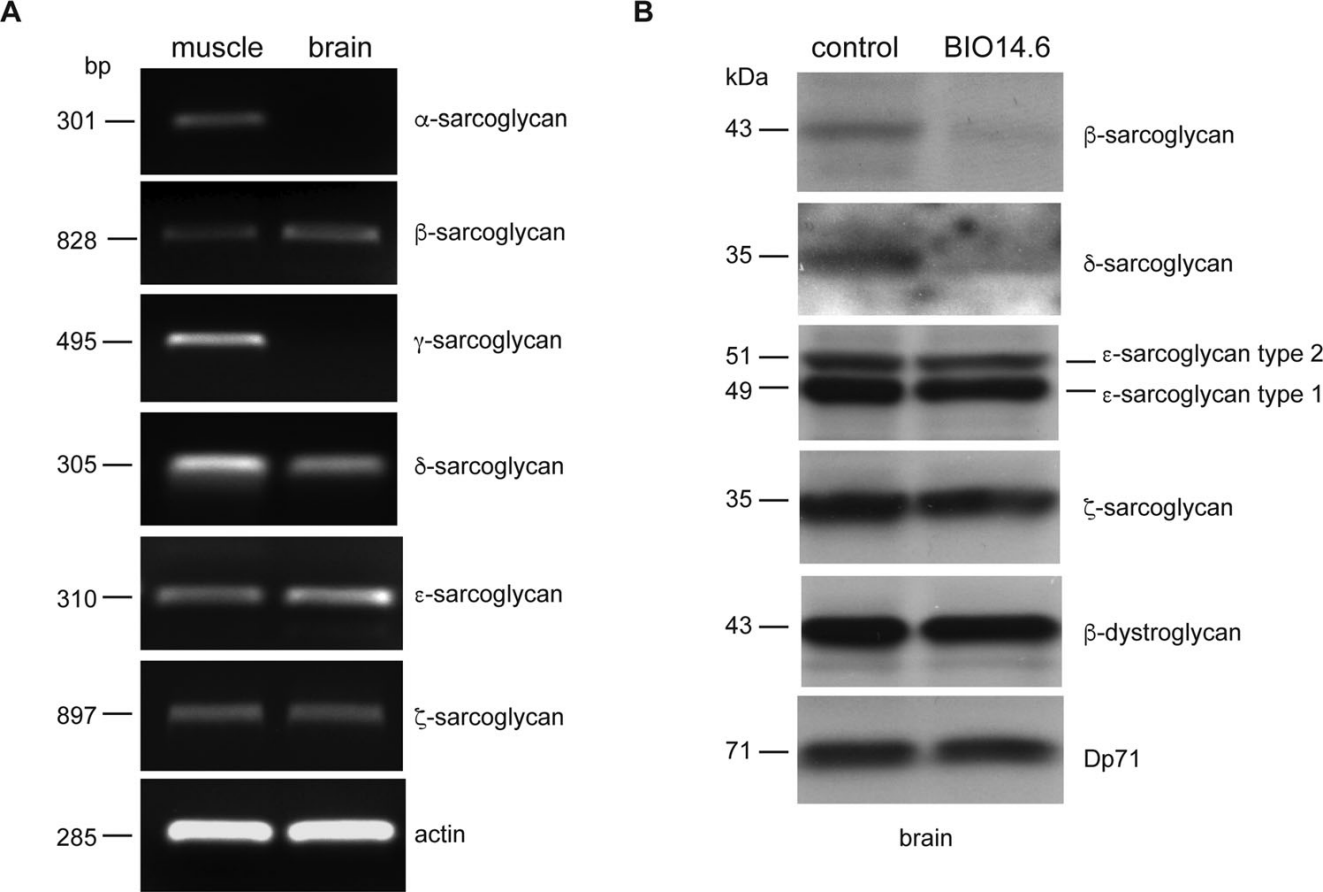
Detection of sarcoglycan transcripts and protein in brain. PCR products were amplified from single‐stranded DNA prepared from RNA extracted from adult rat skeletal muscle and brain (A). PCR primers for amplifying rat sarcoglycan cDNA were described previously.^26^ Sizes of the expected PCR product are shown in base pairs. Note that α‐sarcoglycan and γ‐sarcoglycan transcripts were not detected in brain. Sarcoglycan levels in the BIO14.6 hamster brain (B). Membrane fractions prepared from wild‐type controls and δ‐sarcoglycan‐deficient BIO14.6 brain were analyzed by Western blotting using antibodies against the sarcoglycans (ANA‐ɛ and ANA‐ζ), β‐dystroglycan and Dp71. The absence of δ‐sarcoglycan in the BIO14.6 hamster is associated with a reduced level of β‐sarcoglycan but not ɛ‐ and ζ‐sarcoglycan. Note that the 2 proteins detected with the anti‐ɛ‐sarcoglycan antibodies correspond to the brain‐specific (upper) and ubiquitous ɛ‐sarcoglycan isoforms. The levels of Dp71 and β‐dystroglycan are similarly unaltered in the BIO14.6 hamster compared with the wild‐type control.

Although the involvement of *SGCE* in the genetic etiology of M‐D is well established, little is known about the function of ɛ‐sarcoglycan and the other sarcoglycans in the brain including whether the proteins physically associate to form a stable protein complex. Initially, we used reverse‐transcription PCR to demonstrate the presence of the sarcoglycan transcripts in rat brain. PCR products corresponding to ɛ‐, β‐, δ‐, and ζ‐sarcoglycan were amplified from first‐strand cDNA prepared from brain and muscle RNA. In contrast, α‐ and γ‐sarcoglycan were not readily detected in brain, whereas a product in muscle was clearly resolved (Fig. 1A). These data are in agreement with the original studies on ɛ‐ and ζ‐sarcoglycan showing their widespread expression pattern contrasting with the more restricted tissue distribution of α‐ and γ‐sarcoglycan.^16^, ^26^, ^45^, ^46^

Having demonstrated that some of the sarcoglycans are expressed in the brain, Western blots were used to determine whether a cognate protein was also present. In these experiments we used tissue derived from the cardiomyopathic BIO14.6 hamster. The BIO14.6 hamster carries a mutation in the promoter and first exon of the hamster *Sgcd* gene that in the homozygous state results in adult lethality.^47^, ^48^ Previous studies have shown that the DGC dissociates in the BIO14.6 hamster leading to a compound deficiency of the sarcoglycan complex at the sarcolemma.^48^ Membrane preparations were used to increase the chance of detecting potentially low‐abundance sarcoglycans in the brain. These experiments showed that β‐sarcoglycan and δ‐sarcoglycan were severely reduced in membrane preparations derived from the brains of the BIO14.6 hamster compared with controls (Fig. 1B). In contrast, the levels of ɛ‐sarcoglycan‐1 and ‐2 (see below), ζ‐sarcoglycan, and other components of the DGC (β‐dystroglycan and Dp71) were apparently unaltered in the BIO14.6 hamster brain.

### Sarcoglycan Complexes in Brain

We used immunoaffinity purification (IAP) and mass spectrometry to enrich and identify endogenous ɛ‐sarcoglycan‐containing protein complexes in brain. Esg‐4990‐conjugated protein A agarose beads were used to purify ɛ‐sarcoglycan from rat brain, heart, and lung tissue solubilized in RIPA buffer. Heart and lung contain prototypical tetrameric sarcoglycan complexes and were used for comparative purposes and as a positive control.^33^

**Figure 2:**
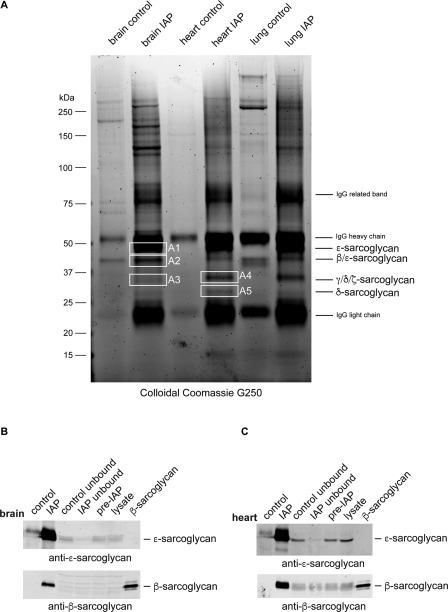
IAP of ɛ‐sarcoglycan containing protein complexes from rat tissue. Esg‐4990‐protein A agarose beads were used to purify ɛ‐sarcoglycan and associated proteins from rat brain, heart, and lung. Proteins eluted from esg‐4990‐protein A agarose beads (IAP) or protein A agarose beads (control) were resolved by SDS‐PAGE using a 4%‐12% bis‐Tris gradient gel and visualized with colloidal Coomassie blue G250 dye (A). Bands (A1‐A5) were excised and processed for mass spectrometry. The identity of the major protein in each gel plug is indicated. Note that the strongly stained bands in each IAP correspond to the IgG heavy and light chains and partially obscure ɛ‐sarcoglycan at 50 kDa. Western blots of protein extracts used for IAP derived from brain (B) and heart (C). Each panel shows the original RIPA‐extract (lysate), the precleared extract (pre‐IAP), the IAP, the flow‐through following IAP (IAP unbound), and the flow‐through from the protein A agarose control. Positive controls (brain extract, ɛ‐sarcoglycan; HEK cells expressing β‐sarcoglycan, β‐sarcoglycan) for each antibody are also shown. As expected, ɛ‐sarcoglycan is highly enriched in the IAP and depleted from the flow‐through in both tissues. Similarly, β‐sarcoglycan is present in the IAP, although in the brain the cognate protein could not be detected in the original RIPA‐extract probably because of its relatively low abundance.

Immunoaffinity‐purified ɛ‐sarcoglycan‐containing complexes were separated by polyacrylamide gel electrophoresis and stained with colloidal Coomassie blue. Bands from each tissue were compared with the appropriate protein‐A bead control. Several proteins copurify with ɛ‐sarcoglycan using esg‐4990‐conjugated beads that were not present in the protein‐A control (Fig. 2A). Initially, we focused on the region between 55 and 25 kDa spanning the size range of all 6 sarcoglycans. Bands were excised as indicated and processed for mass spectrometry as described previously.^49^ After filtering common contaminants such as keratin and immunoglobulins,^50^ the list of protein identities for each band was ranked according to their *P* values in Bioworks (Table 1). Peptides corresponding to all the sarcoglycans except α‐sarcoglycan were identified with high confidence in each experiment from rat brain (Supplementary Table 1). Peptides derived from ɛ‐sarcoglycan‐2 and exon 8^+^ variants were also found by mass spectrometry (Supplementary Table 1). In contrast, ɛ‐sarcoglycan‐1‐specific peptides would be difficult to detect in this experiment because the unique C‐terminal is only 4 amino acids and also contains a lysine residue that would be cleaved during the trypsinization process. In heart, ɛ‐sarcoglycan copurified with β‐, γ‐ and δ‐sarcoglycan, suggesting the presence of a prototypical sarcoglycan complex as described previously.^33^ Although ζ‐sarcoglycan was not identified in the heart IAP, the peptide counts and coverage of the sarcoglycans purified from heart were comparable to those identified in brain (Supplementary Table 1).

**Table 1:** Mass spectrometry statistics for pan‐ɛ‐sarcoglycan immunoaffinity purification from rat brain

Protein	*P*	% Coverage	Peptides	MW
ɛ‐Sarcoglycan	2.22 × 10^−16^	33.2	16	49,808.1
β‐Sarcoglycan	1.11 × 10^−16^	18.3	13	41,643.3
ζ‐Sarcoglycan	8.50 × 10^−13^	25.5	12	32,892.8
γ‐Sarcoglycan	4.28 × 10^−12^	9.6	4	32,159.5
δ‐Sarcoglycan	1.25 × 10^−11^	46.7	21	32,100.9

The table summarizes the peptide sequence coverage of the different sarcoglycans identified in bands A1 to A3 (Fig. 2A) following IAP of ɛ‐sarcoglycan using esg‐4990 that detects all ɛ‐sarcoglycan isoforms. A complete list of peptides identified in these experiments can be found in Supplementary Table 1.

Western blot analysis of the IAP samples with an anti‐ɛ‐sarcoglycan antibody demonstrated enrichment of endogenous ɛ‐sarcoglycan from brain and heart tissue consistent with efficient depletion of ɛ‐sarcoglycan as judged by comparison of the pre‐IAP and post‐IAP lysate samples (Fig. 2B,C). In addition to ɛ‐sarcoglycan, β‐sarcoglycan was detected among the proteins eluted from the esg‐4990 beads, even though, in the case of brain, the levels of β‐sarcoglycan were very low in the homogenate (Fig. 2B,C).

Having shown that a sarcoglycan complex exists in brain using an anti‐ɛ‐sarcoglycan antibody that detects all isoforms, we generated ɛ‐sarcoglycan‐2‐specific antibodies (esg2‐1355 and esg2‐1358) for IAP to determine whether the brain‐specific isoforms were also present in a sarcoglycan complex. These antibodies only detect ɛ‐sarcoglycan‐2 in transfected cells and brain extracts. Both esg2‐1355 and esg2‐1358 effectively immunoprecipitated ɛ‐sarcoglycan from RIPA extracts prepared from mouse brain (Supplementary Fig. 1). Again, Coomassie‐stained bands between 55 and 25 kDa were excised, and proteins were identified using mass spectrometry as described above. In addition to ɛ‐sarcoglycan‐2, peptides corresponding to β‐, δ‐, and ζ‐sarcoglycan were identified with high confidence (Supplementary Table 2). Interestingly, γ‐sarcoglycan was not among the proteins identified in these experiments. These data are consistent with the low abundance of γ‐sarcoglycan in brain and its relatively poor peptide coverage in the esg‐4990 brain IAP compared with heart (Fig. 1 and Supplementary Table 1). It is also possible that γ‐sarcoglycan is derived from the smooth‐muscle sarcoglycan complex found in the brain vasculature.^35^

**Figure 3:**
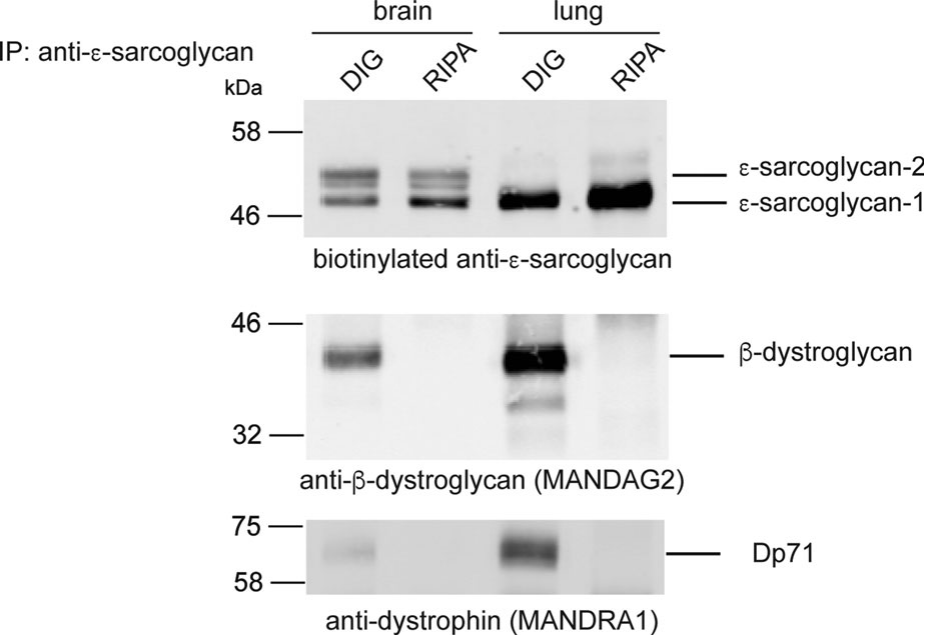
Brain sarcoglycan complex associates with DGC components. Sarcoglycan complexes were immunoaffinity‐purified from mouse brain and lung tissue solubilized in either digitonin‐(DIG) or RIPA (RIPA)‐containing buffers using the pan anti‐ɛ‐sarcoglycan antibody. The biotinylated anti‐ɛ‐sarcoglycan antibody detects ɛ‐sarcoglycan‐1 and ‐2 in brain and ɛ‐sarcoglycan‐1 in lung. β‐Dystroglycan and dystrophin Dp71 only copurify with ɛ‐sarcoglycan in digitonin‐containing buffer. Note that ɛ‐sarcoglycan is more effectively solubilized in RIPA buffer compared with digitonin. Similar results were also obtained using antibodies that recognize only ɛ‐sarcoglycan‐2 (data not shown).

We also used immunoaffinity chromatography to search for other components of the DGC that copurified with the sarcoglycan complex from brain. No peptides corresponding to DGC proteins (other than the sarcoglycans) were identified by mass spectrometry from proteins purified from RIPA extracts of brain, heart, and lung. Although the sarcoglycan complex appeared to be stable in RIPA buffer, we reasoned that other components of the DGC might be lost using stringent buffers. Accordingly, we used 1% digitonin to extract the sarcoglycan complex from brain and lung. Western blotting of immunoaffinity‐purified proteins extracted from brain or lung in either digitonin or RIPA buffer showed copurification of β‐dystroglycan and Dp71 only with digitonin (Fig. 3). Thus, in brain the sarcoglycan complex is associated with other components of the DGC.

### Modeling Brain Sarcoglycan Complex Formation In Vitro

**Figure 4:**
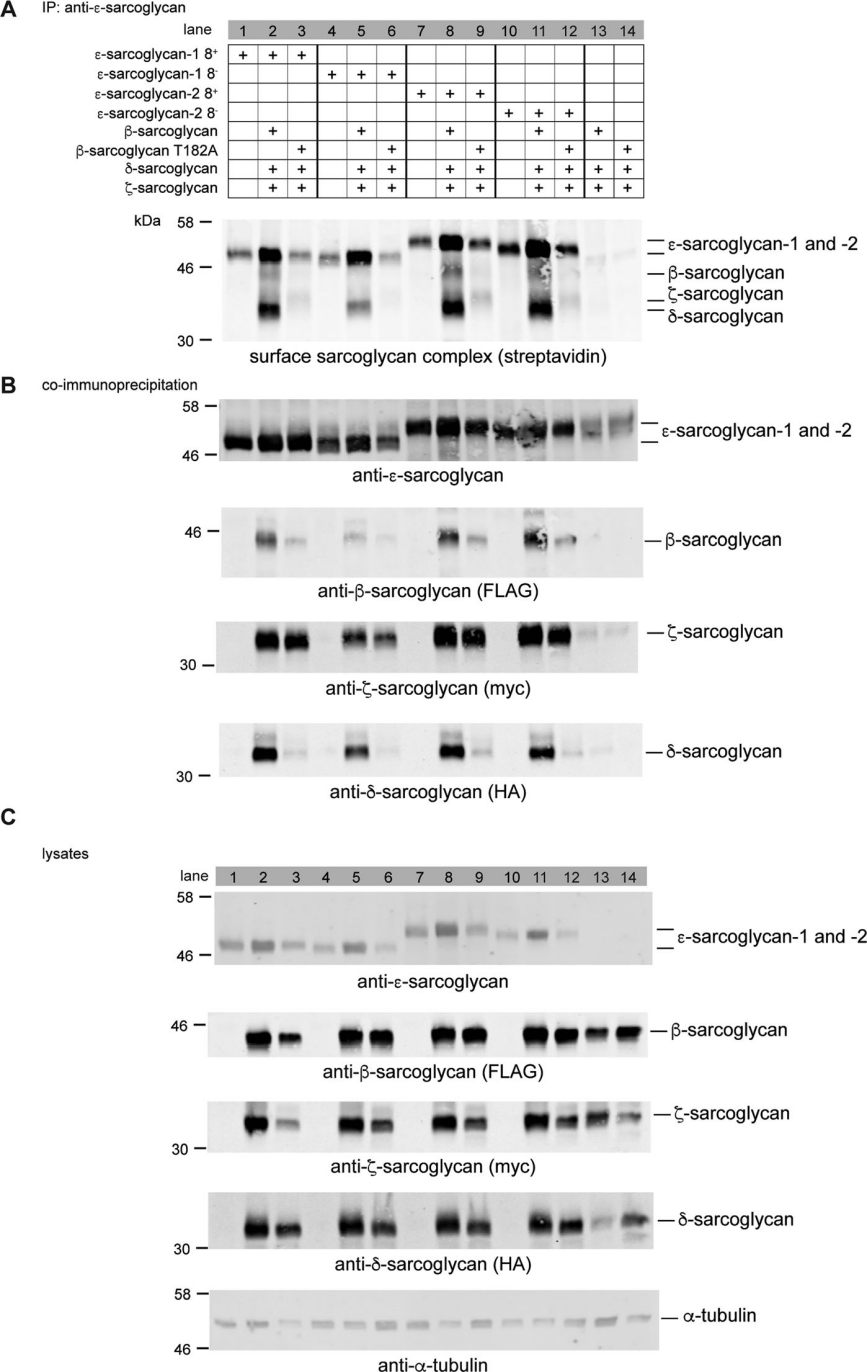
Membrane trafficking of the brain sarcoglycan complex. HEK293T cells were transfected with different combinations of epitope‐tagged sarcoglycans as indicated. After surface biotinylation, esg‐4990 beads were used to immunoprecipitate ɛ‐sarcoglycan and associated proteins. Biotinylated surface proteins that coimmunoprecipitate with ɛ‐sarcoglycan were identified using streptavidin‐Alexa Fluor 680 (A). For comparative purposes, data are presented in groups of 3 (lanes 1‐12) corresponding to the 4 major brain ɛ‐sarcoglycan isoforms. Each group follows the same scheme; ɛ‐sarcoglycan isoform alone, ɛ‐sarcoglycan isoform in a tetramer with wild‐type β‐sarcoglycan and ɛ‐sarcoglycan isoform in a tetramer with mutant β‐sarcoglycan. Lanes 13 and 14 show that trimers that form in the absence of transfected ɛ‐sarcoglycan with either wild‐type or mutant β‐sarcoglycan do not traffic to the cell surface. Although ɛ‐sarcoglycan isoforms traffic to the cell surface in the absence of the other sarcoglycans (lanes 1, 4, 7, and 10), the highest levels of sarcoglycans at the membrane were observed in cells coexpressing ɛ‐, ζ‐, and δ‐sarcoglycan with wild‐type β‐sarcoglycan (lanes 2, 5, 8, and 11). By contrast, levels of δ‐sarcoglycan and, to a lesser extent, ɛ‐ and ζ‐sarcoglycan were drastically reduced in cells expressing the LGMD2E‐associated β‐sarcoglycan T182A mutant (lanes 3, 6, 9, and 12). Complex formation was demonstrated by coimmunoprecipitation for all four ɛ‐sarcoglycan isoforms (B). Robust coimmunoprecipitation of each sarcoglycan is observed for all heterotetramers that contain wild‐type β‐sarcoglycan (lanes 2, 5, 8, and 11). By contrast, βδ‐sarcoglycan core formation was severely disrupted in cells expressing mutant β‐sarcoglycan compared with wild type; however, the association of ɛ‐ and ζ‐sarcoglycan was apparently unaffected by the mutant (lanes 3, 6, 9, and 12). Whole‐cell lysates are shown for comparative purposes, whereas α‐tubulin was used as a loading control (C).

Having shown that a prototypical sarcoglycan complex was present in brain, we examined the assembly of this complex in transfected cells. Surface biotinylation of HEK293T cells cotransfected with different combinations of epitope‐tagged sarcoglycans was used to label the sarcoglycan complex exclusively at the plasma membrane (Fig. 4). After biotinylation, ɛ‐sarcoglycan was immunoprecipitated from the cells using esg‐4990‐protein A agarose beads. Western blotting of the immunoprecipitated proteins with streptavidin‐Alexa Fluor 680 showed that all ɛ‐sarcoglycan isoforms trafficked to the cell surface independently and when coexpressed with the other sarcoglycans (Fig. 4A and Supplementary Fig. 2A). In contrast, β‐ and δ‐sarcoglycan were robustly detected at the cell surface only when coexpressed with ɛ‐ and ζ‐sarcoglycan (Fig. 4, lanes 2, 5, 8, and 11). Although HEK cells express low levels of ɛ‐sarcoglycan (Fig. 2A, lanes 13 and 14), exogenous ɛ‐sarcoglycan is required to promote membrane trafficking of β‐, δ‐, and ζ‐sarcoglycan. These data highlight the importance of the βδ‐sarcoglycan core for plasma membrane deposition of the sarcoglycan complex and also demonstrate that ζ‐sarcoglycan can assemble into a membrane‐associated heterotetramer in heterologous cells as described previously.^29^, ^51^ Each ɛ‐sarcoglycan isoform coimmunoprecipitated with the other sarcoglycans, demonstrating that they were individually capable of assembling into a prototypical heterotetramer (Fig. 4B). Importantly, these findings were also confirmed when NeutrAvidin was used to affinity‐purify biotinylated cell surface proteins (Supplementary Fig. 2A,B).

Previous studies in cell models have shown that sarcoglycan mutants impair trafficking and assembly of the sarcoglycan complex, recapitulating findings in the different sarcoglycanopathies.^51^, ^52^ Therefore, we examined the effect of an endoplasmic reticulum (ER)‐retained β‐sarcoglycan missense mutation (T182A) on sarcoglycan complex formation. The T182A mutation in *SGCB* (c.544A>G) is associated with a Duchenne muscular dystrophy–like presentation and cardiomyopathy.^41^, ^53^ The rationale behind this experiment was to determine whether ER retention of the β‐sarcoglycan mutant would disrupt cell surface localization of the brain sarcoglycan complex in heterologous cells. Based on previous studies in LGMD, a β‐sarcoglycan mutation should abrogate trafficking of the entire heterotetrameric complex, leading to a compound sarcoglycan deficiency at the plasma membrane, as described in LGMD patients. As expected, only residual amounts of β‐sarcoglycan T182A were detected at the plasma membrane using streptavidin‐Alexa Fluor 680 (Fig. 4A) or when surface biotinylated proteins were enriched using neutravidin affinity purification (Supplementary Fig. 2A). However, inclusion of β‐sarcoglycan T182A in the heterotetramer did not abrogate the physical association and membrane trafficking of ɛ‐ and β‐sarcoglycan (Fig. 4A and Supplementary Fig. 2A, lanes 3, 6, 9, and 12). These data recapitulate our findings in the BIO14.6 hamster brain, providing additional support for the notion that in the brain ɛ‐ and ζ‐sarcoglycan may be preserved in the absence of the βδ‐sarcoglycan core.

## Discussion

To better understand the role of ɛ‐sarcoglycan in M‐D, we aimed to establish whether a sarcoglycan complex exists in the brain. We show that ubiquitous and brain‐specific ɛ‐sarcoglycan isoforms copurify with β‐, γ‐, δ‐, and ζ‐sarcoglycan, demonstrating the existence of prototypical sarcoglycan complexes in the brain (Fig. 2 and Supplementary Fig. 1). We also found that the sarcoglycan complex copurifies with other components of the DGC when milder extraction conditions were used to solubilize membrane proteins from brain (Fig. 3).

*Sgce* is expressed primarily in neuronal tissue with some expression in perivascular astrocytes and other types of glial cells.^35^, ^54^ However, a recent study showed that transcripts encoding all of the sarcoglycans were expressed in blood vessels isolated from mouse brain.^55^ Interestingly, only ɛ‐sarcoglycan‐1 has been found in brain‐derived capillary endothelial cells and astrocytes, suggesting that ɛ‐sarcoglycan‐2 may be the predominant ɛ‐sarcoglycan isoform in neurons.^35^ Our failure to detect γ‐sarcoglycan when the ɛ‐sarcoglycan‐2‐specific antibody (esg2‐1358) was used to purify the SGC from brain combined with the low‐peptide coverage of γ‐sarcoglycan when the pan ɛ‐sarcoglycan antibody (esg‐4990) was used for IAP could suggest that γ‐sarcoglycan is derived from nonneuronal cells such as perivascular astrocytes or brain endothelial cells (Table 1 and Supplementary Table 2). These data are consistent with association of multiple DGC‐like complexes in different cell types of the brain.^56^

The presence of a sarcoglycan complex in the brain raises questions about the importance of these associations for M‐D and LGMD. Both M‐D and LGMD are caused by loss‐of‐function mutations leading to a deficiency of the individual sarcoglycans or sarcoglycan complex at the plasma membrane.^12^, ^13^ However, differences in the presentation of these disorders cannot be fully explained based on existing knowledge of the sarcoglycan assembly and function. One possible explanation for these differences is that the early onset and severity of LGMD may mask the features of dystonia in patients with sarcoglycan mutations. Alternatively, partial preservation of the sarcoglycan complex in the brains of some LGMD patients may be sufficient to maintain normal neurological function.

Cai and colleagues showed that the Schwann cell sarcoglycan complex, in common with the brain complex described here, was composed of a ɛβδζ‐sarcoglycan heterotetramer.^26^ Notably, the low abundance of α‐ and γ‐sarcoglycan in nerve was also evident in brain (Fig. 1). Despite these similarities, Cai and coworkers found that the levels of both ɛ‐ and ζ‐sarcoglycan were severely reduced in Schwann cell membrane preparations from δ‐sarcoglycan‐deficient BIO14.6 hamster, contrasting markedly with our findings in brain (Fig. 1B). Although this may seem paradoxical, cytoplasmic components of the DGC were also preserved in the brains of the *Dmd* ^*mdx*^ and *Dmd* ^*mdx3Cv*^ mouse models of Duchenne muscular dystrophy when they were severely reduced in muscle.^56^ Thus, similar sarcoglycan complexes in nerve, muscle, and brain appear to be differentially sensitive to the absence of specific sarcoglycans or other components of the DGC.

To examine this effect in cells, we used transfection to model the well‐documented membrane‐trafficking defect associated with most sarcoglycan mutants by substituting β‐sarcoglycan in a recombinant ɛβδζ tetramer with an LGMD2E‐associated missense mutation (T182A). Previous studies have demonstrated the importance of the βδ‐sarcoglycan core in the assembly and membrane trafficking of the sarcoglycan complex in transfected cells.^51^, ^57^ Thus, a mutation in β‐sarcoglycan should abolish or attenuate membrane trafficking of the entire sarcoglycan complex. Interestingly, we found that the β‐sarcoglycan mutant affected trafficking and assembly of the βδ‐sarcoglycan core but did not abolish membrane trafficking and association of ɛ‐ and ζ‐sarcoglycan (Fig. 4 and Supplementary Fig. 2).

These findings could potentially explain the apparent lack of dystonia‐associated features in LGMD patients as follows. Although ɛ‐sarcoglycan forms a canonical DGC‐associated sarcoglycan complex in brain, it is able to traffic to the membrane with ζ‐sarcoglycan independently of the βδ‐sarcoglycan core. Mutations in either core component do not abrogate the association of ɛ‐ and ζ‐sarcoglycan and its trafficking to the plasma membrane. Thus, and in contrast with the other sarcoglycans in LGMD, a pool of ɛ‐ and ζ‐sarcoglycan in brain evades the quality‐control mechanism that mitigates anterograde trafficking of a mutant sarcoglycan complex.^12^, ^22^, ^24^, ^25^ We also found evidence that higher levels of brain‐specific ɛ‐sarcoglycan were deposited at the membrane compared with ɛ‐sarcoglycan‐1 (Fig. 4). These data may be important in the context of M‐D because even residual levels of the sarcoglycan complex have been shown to be physiologically important in muscle.^58^

From a clinical perspective, our data provide the first evidence that M‐D may be directly associated with the function of the DGC in the brain, as previously postulated. In addition to its well‐characterized role in striated muscle, the DGC has a critical role during brain development.^18^, ^59^ Although the function of the DGC in brain is far from clear, abnormal synaptic electrophysiology, Ca^2+^ homeostasis, and intellectual and behavioral impairments have been documented in patients and preclinical models of muscular dystrophy.^18^, ^60^ Altered neuronal Ca^2+^ homeostasis may provide a mechanistic link between M‐D, the muscular dystrophies, and other forms of dystonia that converge on this pathway. Interestingly, the paralogous genes *ANO3* and *ANO5* that encode putative Ca^2+^‐activated Cl^‐^ channels are mutated in autosomal dominant craniocervical dystonia (DYT23) and LGMD type 2L, respectively.^61^, ^62^, ^63^ Taken together, these data may suggest that abnormal Ca^2+^ signaling may play a broader role in the molecular pathogenesis of dystonia than previously thought.^64^ Our data also suggest that restoration of the complete SGC in cell models of M‐D, including patient‐derived induced pluriopotent stem cells, may be a useful translational biomarker for preclinical studies on M‐D. It is also tempting to speculate that alterations in Ca^2+^ signaling in M‐D and other dystonias may affect network activity and functional connectivity of different brain regions.

In conclusion, we have shown that prototypical sarcoglycan complexes containing different ɛ‐sarcoglycan isoforms exists in brain. Our data and the clinical findings in patients with M‐D and LGMD suggest that a pool of ɛ‐sarcoglycan may traffic and function independently of the core sarcoglycan complex in the brain. These data contribute to our understanding of the role of the sarcoglycans in disease and potentially explain the apparent absence of neurological features in muscular dystrophy patients with sarcoglycan mutations.

## Author Roles

1. Research Project: A. Conception, B. Organization, C. Execution; 2. Manuscript Preparation: A. Writing the First Draft, B. Review and Critique.

A.J.W.: 1A, 1B, 1C, 2A, 2B

F.A.C.: 1C, 2A, 2B

Y.M.C.: 1C, 2B

D.J.B.: 1A, 1B, 2A, 2B

## Supporting information

Additional supporting information may be found in the online version of this article at the publisher's web‐site.

**SUPPLEMENTARY FIG. 1**. IAP of ɛ‐sarcoglycan‐2 from mouse brain. Mouse brain proteins eluted from esg2‐1355 and esg2‐1358‐conjugated protein A beads or protein A beads only were resolved by SDS‐PAGE using a 4%‐12% bis‐Tris gradient gel and visualized with colloidal Coomassie blue G250 dye. Bands B1‐B4 were excised and processed for mass spectrometry. Proteins were eluted sequentially in sample buffer (E1) and sample buffer with 50 mM DTT at 95 °C (E2) to minimize IgG leaching from the affinity matrices. The identity of the proteins found in each gel plug is indicated.

**SUPPLEMENTARY FIG. 2**. Assessing membrane trafficking of the brain sarcoglycan complex using NeutrAvidin capture. NeutrAvidin capture of membrane‐localized sarcoglycans in heterologous cells (A). The figure is arranged following the scheme detailed in Figure 4. HEK293T cells were transfected with different combinations of epitope‐tagged sarcoglycans as indicated. After surface biotinylation, NeutrAvidin agarose beads were used to immunoprecipitate biotinylated membrane proteins. Although ɛ‐ and ζ‐sarcoglycan can be detected at the membrane, coexpression of all 4 sarcoglycans promotes robust trafficking of the wild‐type sarcoglycan tetramer (lanes 2, 5, 8, and 11). By contrast, in cells expressing the LGMD2E‐associated β‐sarcoglycan T182A mutant, the levels of βδ‐sarcoglycan core were severely reduced at the cell surface (lanes 3, 6, 9, and 12). Importantly, membrane trafficking of ɛ‐ and ζ‐sarcoglycan was apparently unaffected by the T182A mutant. Whole‐cell lysates are shown for comparative purposes whereas α‐tubulin was used as a loading control (B).

Supplementary Information Table 1.

Supporting Information Table 2.
