# Supplementary figures and images for: Myoclonus dystonia and muscular dystrophy: ɛ‐sarcoglycan is part of the dystrophin‐associated protein complex in brain
Source: Mov Disord. 2016 Aug 18;31(11):1694–703. doi: 10.1002/mds.26738 (PMC5129563; doi:10.1002/mds.26738)

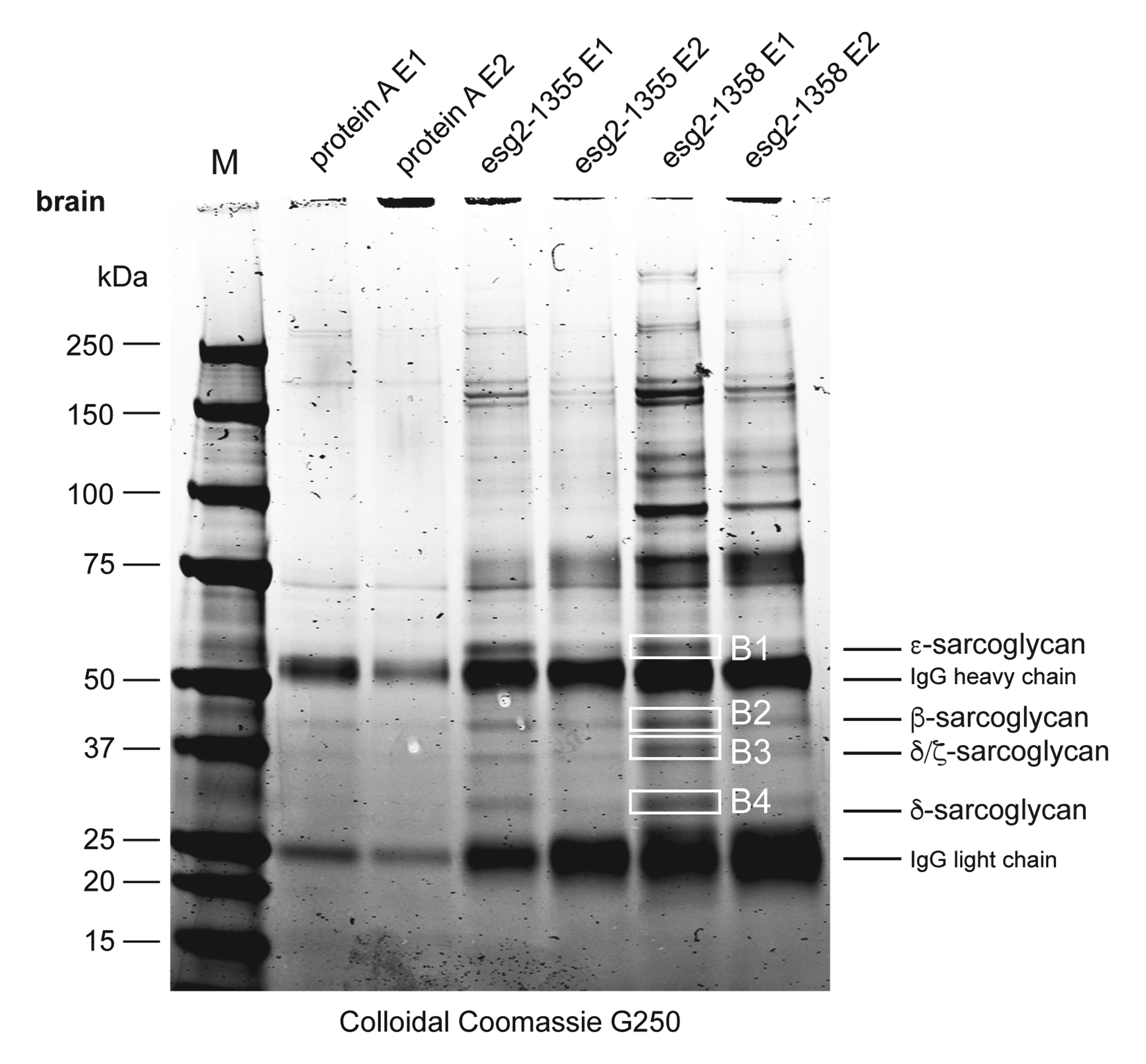

Supplement: Supplementary file 1 — SUPPLEMENTARY FIG. 1. IAP of ɛ‐sarcoglycan‐2 from mouse brain. Mouse brain proteins eluted from esg2‐1355 and esg2‐1358‐conjugated protein A beads or protein A beads only were resolved by SDS‐PAGE using a 4%‐12% bis‐Tris gradient gel and visualized with colloidal Coomassie blue G250 dye. Bands B1‐B4 were excised and processed for mass spectrometry. Proteins were eluted sequentially in sample buffer (E1) and sample buffer with 50 mM DTT at 95 °C (E2) to minimize IgG leaching from the affinity matrices. The identity of the proteins found in each gel plug is indicated. [file MDS-31-1694-s001.tif]

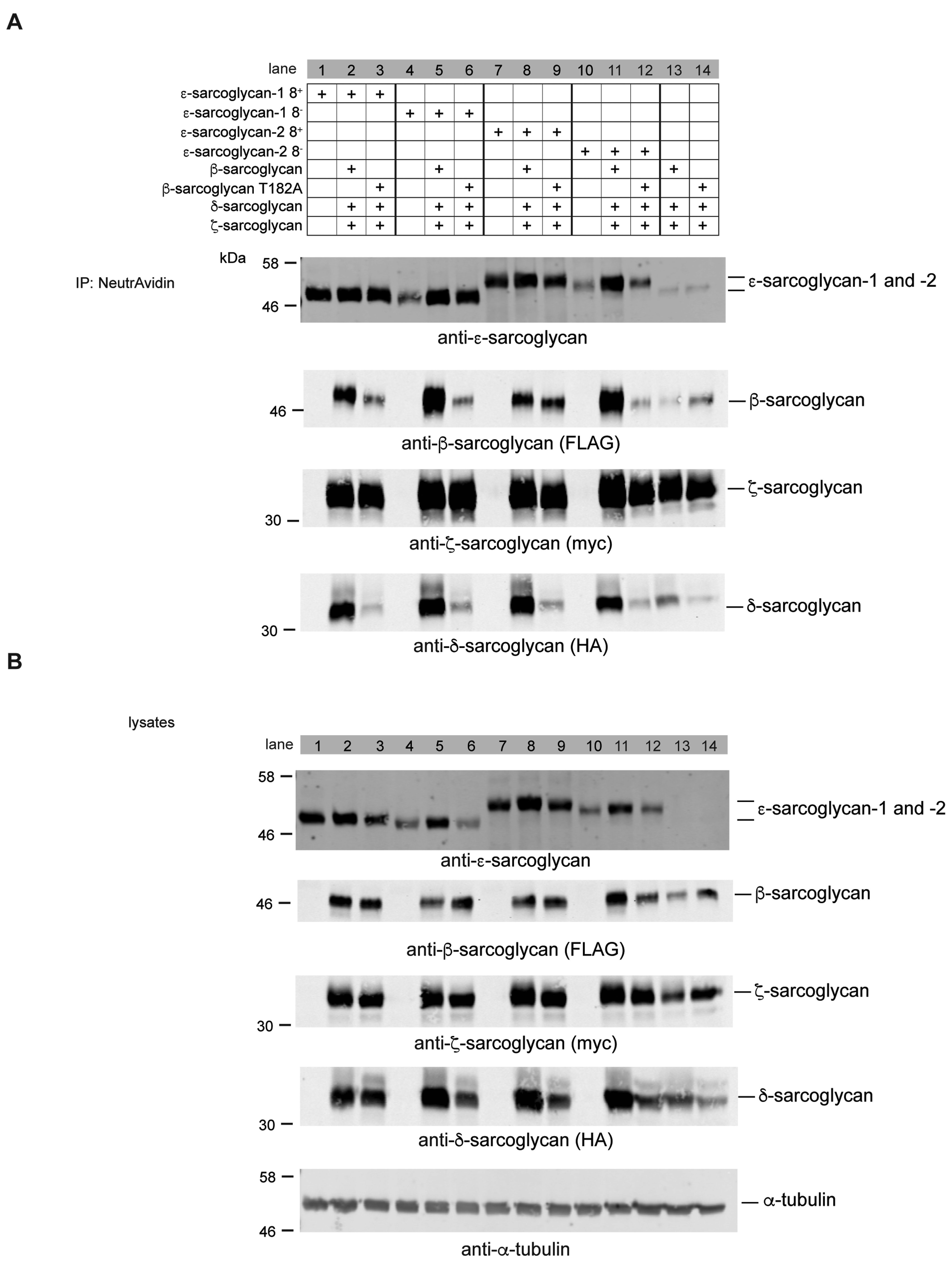

Supplement: Supplementary file 2 — SUPPLEMENTARY FIG. 2. Assessing membrane trafficking of the brain sarcoglycan complex using NeutrAvidin capture. NeutrAvidin capture of membrane‐localized sarcoglycans in heterologous cells (A). The figure is arranged following the scheme detailed in Figure 4. HEK293T cells were transfected with different combinations of epitope‐tagged sarcoglycans as indicated. After surface biotinylation, NeutrAvidin agarose beads were used to immunoprecipitate biotinylated membrane proteins. Although ɛ‐ and ζ‐sarcoglycan can be detected at the membrane, coexpression of all 4 sarcoglycans promotes robust trafficking of the wild‐type sarcoglycan tetramer (lanes 2, 5, 8, and 11). By contrast, in cells expressing the LGMD2E‐associated β‐sarcoglycan T182A mutant, the levels of βδ‐sarcoglycan core were severely reduced at the cell surface (lanes 3, 6, 9, and 12). Importantly, membrane trafficking of ɛ‐ and ζ‐sarcoglycan was apparently unaffected by the T182A mutant. Whole‐cell lysates are shown for comparative purposes whereas α‐tubulin was used as a loading control (B). [file MDS-31-1694-s002.tif]
