# Supplementary material for: Myoclonus dystonia and muscular dystrophy: ɛ‐sarcoglycan is part of the dystrophin‐associated protein complex in brain
Source: Mov Disord. 2016 Aug 18;31(11):1694–703. doi: 10.1002/mds.26738 (PMC5129563; doi:10.1002/mds.26738)
Supplement: Supplementary file 3 — Supplementary Information Table 1. [file MDS-31-1694-s003.docx]

**Supplementary Table 1 Peptide identification data from immunoaffinity purification of ε-sarcoglycan from rat brain and heart.**

| **Band** | **Identification** | **Peptides** | ***P* (Pro)** | **Coverage** | **MW** |
| --- | --- | --- | --- | --- | --- |
| A1  (Brain) | **ε-sarcoglycan**  gi\|50300481\|ref\|NP_001002023.1\| sarcoglycan, epsilon [Rattus norvegicus] | R.CSQEmEPVITCDKK.F  R.EVENPQNQLR.C  R.GGRVPLPIKDmK.E  K.NVWQPER.L  R.VPLPIKDmK.E  K.QVSTYQEVVR.G  **R.NmQTPDIQLVHHSSIQK.S^1^**  -.GEGILPDGGEYKPPSDSLK.-  R.GEGILPDGGEYKPPSDSLK.S  R.TQFYIDWCK.I  R.TPYSDGVLYGSPTAENVGKPTIIEITAYNRR.T  R.TPYSDGVLYGSPTAENVGKPTIIEITAYNR.R  -.NVYPSAGVLFVHVLER.-  R.NVYPSAGVLFVHVLER.E | 2.22E-16 | 33.2 | 49.8 |
| A1  (Brain) | **ε-sarcoglycan (type-2)** | **R.LTTFQR.F^2^**  **R.FEVnGIPEER.Q^2^** | 8.57E-5 | 3.6 | 51.3 |
| A2  (Brain) | **β-sarcoglycan**  gi\|109499619\|ref\|XP_001056227.1\| PREDICTED: similar to β-sarcoglycan (Beta-SG) (43 kDa dystrophin-associated glycoprotein) (43DAG) [Rattus norvegicus] | R.GNEGVFImGK.T  R.LPSSSGGDQSGSGDWVR.Y  K.AGYIPIDEDR.L  K.AGYIPIDEDRLHK.T  R.GNEGVFIMGK.T  R.RNENLVITGNNQPIVFQQGTTK.L  -.RNENLVITGnNQPIVFQQGTTK.-  R.RnENLVITGNNQPIVFQQGTTK.L | 1.11E-16 | 18.3 | 41.6 |
| A2  (Brain) | **ε-sarcoglycan**  gi\|50300481\|ref\|NP_001002023.1\| sarcoglycan, epsilon [Rattus norvegicus] | R.EVENPQNQLR.C  K.NVWQPER.L  K.QVSTYQEVVR.G  R.NmQTPDIQLVHHSSIQK.S  R.GEGILPDGGEYKPPSDSLK.S  R.TQFYIDWCK.I | 8.86E-06 | 16.5 | 49.8 |
| A3  (Brain) | **ζ-sarcoglycan**  gi\|149057986\|gb\|EDM09229.1\| sarcoglycan zeta (predicted) [Rattus norvegicus] | R.SLImEAPR.G  R.GVQVSAAAGDFK.A  R.SLIMEAPR.G  K.VTGTEGAVFGHSVETPHVR.A  R.LGNLPIGSFSSSPSSSNSR.Q  R.LGNLPIGSFSSSPSSSnSR.Q  -.LGnLPIGSFSSSPSSSNSR.-  R.VLFSADEDEITIGAEK.L  R.VLFSADEDEITIGAEKLK.V | 8.50E-13 | 25.5 | 32.9 |
| A3  (Brain) | **γ-sarcoglycan**  gi\|55741776\|ref\|NP_001006994.1\| gamma sarcoglycan [Rattus norvegicus] | R.AEHPFQDLR.L  R.VTGPEGALFEHSVETPLVR.A | 4.28E-12 | 9.6 | 32.2 |
| A3  (Brain) | **δ-sarcoglycan**  gi\|198386317\|ref\|NP_001128298.1\| delta-sarcoglycan b [Rattus norvegicus] | R.SLVmEAPK.G  R.STmPSSEGPHIYK.V  K.SIETPNVR.A  K.DGEIKLDAAK.I  K.GVEINAEAGNmEATCR.S  R.SLVMEAPK.G  K.SRPGNALYFK.S  R.ADPFKELR.L  K.VLTQLVTGPK.A  R.VLGAEGTVFPK.S  R.LRVLGAEGTVFPK.S  R.ADPFKELRLESPTR.S  K.LLFSADDSEVVVGAER.L  K.LEGDSEFLQPLYAK.E  -.GLKLEGDSEFLQPLYAK.-  K.GLKLEGDSEFLQPLYAK.E | 1.25E-11 | 46.7 | 32.2 |
| A3  (Brain) | **ζ-sarcoglycan**  gi\|171543877\|ref\|NP_001102345.2\| sarcoglycan zeta [Rattus norvegicus | R.STDLDIQELK.M | 1.97E-04 | 3.2 | 34.4 |
| A4  (Heart) | **γ-sarcoglycan**  gi\|55741776\|ref\|NP_001006994.1\| gamma sarcoglycan [Rattus norvegicus] | R.SLSmDAPR.G  -.EQYTTVTEGTHIERPENQHVYK.-  R.AEHPFQDLR.L  K.LKQGTQGPPGSSnGVYEICACPDGK.L  .LKQGTqGPPGSSNGVYEICACPDGK.-  K.LYLSmAGEATTCEEHSHICL.-  K.IGIYGWR.K  R.VTGPEGALFEHSVETPLVR.A  R.LRVTGPEGALFEHSVETPLVR.A  R.LEGESEFLFPLYAK.E | 4.12E-15 | 43.2 | 32.2 |
| A4  (Heart) | **δ-sarcoglycan**  gi\|198386317\|ref\|NP_001128298.1\| delta-sarcoglycan b [Rattus norvegicus] | R.GSYTPTGTR.Q  R.STmPSSEGPHIYK.V  R.SLVmEAPK.G  K.SIETPNVR.A  -.DGEIKLDAAK.-  K.DGEIKLDAAK.I  K.GVEINAEAGNmEATCR.S  -.STMPSSEGPHIYK.-  R.SLVMEAPK.G  K.SRPGNALYFK.S  R.ADPFKELR.L  K.VLTQLVTGPK.A  K.VFEVCVCAnGR.L  R.VLGAEGTVFPK.S  K.VmNFTIDGmGNLR.I  K.VGIYGWR.K  K.LLFSADDSEVVVGAER.L  K.LEGDSEFLQPLYAK.E  -.GLKLEGDSEFLQPLYAK.- | 4.50E-13 | 43.2 | 32.1 |
| A5  (Heart) | **δ-sarcoglycan**  gi\|198386317\|ref\|NP_001128298.1\| delta-sarcoglycan b [Rattus norvegicus] | R.SLVmEAPK.G  K.VLTQLVTGPK.A  R.VLGAEGTVFPK.S  K.LLFSADDSEVVVGAER.L | 1.72E-10 | 15.6 | 32.1 |

The table shows the peptide sequence data derived from proteins in bands A1 to A5 (Fig. 2A) following immunoaffinity purification of ε-sarcoglycan using esg-4990. The list was filtered to remove probable false-positives. Peptides in bold text are derived from alternatively spliced exons, inclusion of exon 8^1^ and inclusion of exon 11b^2^ (ε-sarcoglycan-2). Lower case residues indicate the following post-translational modifications; oxidation (M), deamidation (N) and (Q).
