# Supplementary material for: Myoclonus dystonia and muscular dystrophy: ɛ‐sarcoglycan is part of the dystrophin‐associated protein complex in brain
Source: Mov Disord. 2016 Aug 18;31(11):1694–703. doi: 10.1002/mds.26738 (PMC5129563; doi:10.1002/mds.26738)
Supplement: Supplementary file 4 — Supporting Information Table 2. [file MDS-31-1694-s004.docx]

**Supplementary Table 2 Peptide identification data from ε-sarcoglycan-2 immunoaffinity purification from mouse brain.**

| **Band** | **Identification** | **Peptides** | **XC Score** | **Coverage** | **MW** |
| --- | --- | --- | --- | --- | --- |
| B1  (Brain) | **ε-sarcoglycan**  gi: 47847004  brain epsilon-sarcoglycan [Mus musculus] | TPYSDGVLYGSPTAENVGKPTIIEITAYNR  NVYPSAGVLFVHVLER  QVSTYQEVVR  GEGILPDGGEYKPPSDSLK  **FEVnGIPEER**  NmNVEEmLASEVLGDFLGAVK  THFHIDWcK  **FEVNGIPEER**  VPLPINDMK  EVENPQnQLR  eVENPQNQLR  TPYSDGVLYGSPTAENVGKPTIIEITAYNRR  EVENPQNQLR  VPLPInDmK  **KLTEAMSL**  NVWQPER  VPLPINDmK | 302.62 | 35.13% | 48.4kDa |
| B2  (Brain) | **β-sarcoglycan**  gi: 6755482  beta-sarcoglycan [Mus musculus] | aAAAAAAAATEQQGSnGPVKK  RNENLVITGNNQPIVFQQGTTK  THNILFSTDYETHEFHLPSGVK  TSITSDIGmQFFDPR | 76.08 | 25.00% | 34.9kDa |
| B3  (Brain) | **δ-sarcoglycan**  gi: 6755484  delta-sarcoglycan [Mus musculus] | LLFSADDSEVVVGAER  LEGDSEFLQPLYAK  VLTQLVTGPK  VLGAEGTVFPK  SIETPNVR  SRPGNALYFK  SLVmEAPK | 96.54 | 26.65% | 32.1kDa |
| B3  (Brain) | **ζ-sarcoglycan**  gi: 148703539  zeta-sarcoglycan [Mus musculus] | VLFSADEDEITIGAEK  STDLDIQELK | 88.30 | 10.09% | 25.2kDa |

The table shows peptide signatures of proteins excised from bands B1 to B4 (Supplementary Fig. 1) from ε-sarcoglycan immunoaffinity purification using esg2-1358, following removal of probable false-positives. XC is the cross correlation score. Lower case residues indicate the following post-translational modifications; oxidation (M), deamidation (N), carbamidomethylation (C) and N-terminal acylation (E and A). Peptides in bold type originate from the unique C-terminus of ε-sarcoglycan-2.
